# Patient and Physician Narratives on Collaborative Care Between Emergency and Family Medicine Services: A Narrative Review

**DOI:** 10.7759/cureus.98099

**Published:** 2025-11-29

**Authors:** Ejike H Anyaegbu, Quadri A Sanni, Adaorah C Mbomi, Kingsley C Obiajunwa, Joseph O Ogbeifun, Ebenezer Akore Yeboah

**Affiliations:** 1 Emergency Medicine, South Warwickshire University National Health Service (NHS) Foundation Trust, West Midlands, GBR; 2 Urology, South Warwickshire University National Health Service (NHS) Foundation Trust, West Midlands, GBR; 3 Family Medicine, National Health Service (NHS) England Workforce, Training and Education Directorate – Wessex, Dorset, GBR; 4 Emergency Medicine, King Khalid Hospital, Najran, SAU; 5 Intensive Care Unit, King Khalid Hospital, Najran, SAU; 6 Institute for Health and Wellbeing, Coventry University, Coventry, GBR

**Keywords:** collaborative care, emergency medicine, family medicine, health services, patient outcome

## Abstract

Interprofessional collaboration between emergency medicine (EM) and family medicine (FM) is essential to ensure continuity, quality, and safety of patient care. While much has been written about system-level integration, fewer studies have focused on the lived experiences and narratives of patients and physicians navigating care transitions between these services. This narrative review synthesizes qualitative and mixed-method studies that explore the perspectives of both physicians and patients on collaborative care between EM and FM. A thematic analysis of selected literature reveals recurring themes of communication challenges, role ambiguity, care fragmentation, and emotional burden. While some narratives highlight positive, coordinated experiences, others describe systemic barriers that compromise patient outcomes and satisfaction. Gaps in the literature include limited patient voices and a lack of longitudinal perspectives. This review underscores the need for more inclusive, story-driven research to inform collaborative practice models that are both clinically effective and experientially meaningful.

## Introduction and background

Collaborative care in the context of emergency medicine (EM) and family medicine (FM)

In the context of emergency medicine, effective collaboration and communication between paramedics and medical staff are crucial for ensuring the safe and seamless handover of patients, ultimately enhancing patient care and outcomes [1]. The handover of care is a pivotal moment in the patient journey, where effective communication and collaborative practice are paramount in ensuring patient safety and preventing medical errors [1]. Collaborative care is a patient-centered healthcare model that tackles behavioural health and substance use issues built on the chronic care management framework. Collaborative care combines key elements like brief behavioural interventions, team-based care coordination, evidence-based pharmacotherapy, and long-term care management. This approach ensures comprehensive and coordinated healthcare delivery [2,3].

Within family medicine settings, Noel et al. (2005) [4] and von Korff et al. (1997) [3] proposed a shift from disease-specific models to patient-centred collaborative care. This approach consists of four key components; collaborative problem definition through identifying patient-defined issues alongside medical diagnoses, targeted goal setting and planning by setting realistic objectives and developing action plans tailored to patient preferences and readiness, self-management training and support services by providing patients with skills, guidance, and emotional support for health behaviour changes, active and sustained follow-up through regular monitoring, complication identification, and progress reinforcement. Von Korff argued that these elements form a core set of services for chronic illness care that can be applied across diseases, eliminating the need for redundant development [3,4]. 

Effective care emerges from the dynamic interaction between patients and clinicians, where they engage in collaborative conversation to develop a shared understanding of the patient’s challenges. Together, they explore and identify solutions that cater to the patient’s unique priorities and goals [5]. This collaborative approach is at the heart of shared decision-making, where patients and clinicians work together to weigh the benefits and risks of different care options and select the best course of action [5,6].

This review aimed to explore the intricacies of collaborative care by examining the experiences and perceptions of patients and physicians in emergency and family medicine services. It sought to understand how these two critical areas of healthcare work together, identifying hurdles to effective communication and collaboration, as well as factors that facilitate smoother interactions. The review also examined how collaborative efforts can lead to improved patient outcomes, reduced medical errors, and enhanced care quality. Furthermore, it aimed to inform strategies to bolster collaboration and communication, potentially leveraging tools like electronic health records. Ultimately, the review sought to contribute to a deeper understanding of how collaborative care models can be optimised for better healthcare delivery, including their impact on patient satisfaction, healthcare resource utilisation, and associated costs.

## Review

Methodology 

The search strategy involved a comprehensive search of multiple databases, including PubMed, Scopus, CINAHL, Embase and grey literature, using specific search terms such as "emergency medicine", "family medicine", "interprofessional collaboration", "narratives", "qualitative", and "patient experience". Studies were included if they focused on the perspectives of patients or physicians, explored interprofessional collaboration between emergency medicine and family medicine, and employed qualitative, narrative, or mixed-method designs. Quantitative-only studies and those unrelated to collaboration were excluded. The selection process involved screening and selecting articles based on these criteria, aiming to identify relevant studies that could inform the understanding of collaborative care between emergency and family medicine services.

Thematic synthesis

Communication and Continuity of Care 

Physician Perspectives on Handovers and Coordination: Poor communication is a widespread issue across healthcare settings, particularly in high-stakes situations like patient hand-offs, where swift and effective management is crucial [7]. To address communication barriers, healthcare professionals need efficient strategies that convey comprehensive information while minimizing errors and fostering collaboration [7,8]. The SBAR (Situation, Background, Assessment, Recommendation) tool meets this need, providing a structured framework for effective communication and handovers [7]. Widely used in various healthcare settings, SBAR promotes clear and logical information sharing, both within and across professions [7,9]. Its structured format enables preparation, ensures relevant details are covered, and enhances understanding between senders and receivers, ultimately reducing errors and improving collaboration [7,10]. Other barriers to effective handovers include contextual factors like workflow processes and personal factors such as power dynamics, expectations, and unclear responsibilities. These complex and context-dependent issues have not been thoroughly examined in emergency medicine [11].

Patient Experiences of Fragmented or Seamless Care: A fragmented care system can have serious consequences, as seen in the case of a patient with long-term needs. A good example is drawn from a study by Shepherd S (2016), where a woman in 2004 with systemic lupus erythematosus (SLE) visited a new general practitioner (GP) with urinary tract infection symptoms [12]. Despite having a complex medical history, the GP referred her to a private diagnostic service that lacked access to her hospital records. The specialist treated her without considering her unique needs, leading to a misdiagnosis and delayed treatment. Only during a regular SLE review with a multidisciplinary team, including a renal specialist, did the patient receive appropriate care. This case underscores the importance of whole-person, patient-centered care, particularly for individuals with long-term conditions like SLE, to ensure tailored treatment and better health outcomes [12]. 

Individuals with chronic illnesses often experience fragmented care due to their need for ongoing, lifelong treatment [13]. This can lead to gaps in care, redundant services, and inconsistent treatment across different healthcare providers and institutions. Fragmented care is associated with poor patient outcomes, including increased healthcare utilization and higher mortality rates [13,14].

Another case of challenges of fragmented care was also adapted from Shepherd S [12]. A patient with rheumatological needs on immunosuppression therapy for sinusitis was referred to the Ear, Nose, and Throat (ENT) services at her preferred NHS Trust [12]. A patient with immunosuppression due to a long-term condition initially received coordinated care within a single trust, with effective communication between her rheumatologist, immunologist, and ENT specialist. However, when her ENT issue recurred, care was transferred to a new trust, leading to fragmentation and delays. A junior doctor without access to her records managed her case, causing concern and a six-week wait to get added to the ENT waiting list at her original hospital [12].

Trust and Role Clarity

How do Patients and Providers Understand the Roles of EM vs FM: Patients’ choices of primary medical care services are influenced by various factors, including financial status, lifestyle, and sociodemographic profile [15,16]. The availability, accessibility, and affordability of community-based services play a significant role in determining whether patients use emergency departments (EDs). When services are inaccessible or unaffordable, patients often turn to the ED for care. Individuals with chronic and complex conditions frequently rely on hospital care due to a lack of coordinated community-based service, resulting in higher costs, ED overcrowding, and inadequate addressing of underlying health and social issues [15,17].

Tensions or Synergies Between Specialties

Successful care transitions from the ED to primary care are crucial for ensuring quality, patient safety, and cost-effectiveness. Timely transitions are particularly important, as patients are vulnerable to adverse events during this period [18,19]. Follow-up with primary care providers improves disease prevention, monitoring, and self-management of acute and long-term conditions. It also supports ongoing clinical management of conditions addressed during ED visits. However, failed care transitions can lead to issues such as poor understanding of discharge instructions, medication non-adherence, and inadequate self-management [19,20].

Early follow-up with primary care providers can help address these challenges by promoting medication adherence, providing education, and guiding patients through the healthcare system [19,21]. Many European countries have recognised the need to reorganise out-of-hours services to balance patient demand with quality, safety, and satisfaction for both patients and healthcare professionals [22,23]. In Belgium, out-of-hours primary care initially relied on local general physician organisations, known as “circles” which used rotation systems [23]. Since 2003, organized duty centres and walk-in clinics have been developed with public funding, aiming to improve working conditions by rationalising on-call duties and reducing home visits. Some centres are strategically located near hospital emergency services, helping to reduce overcrowding by providing primary care to those without a GP or when their GP is unavailable [23]. These centres also enable emergency departments to refer patients with primary care needs. In some regions, a single contact phone number (1733) has been introduced to centralise and regulate out-of-hours calls according to GP guidelines [23]. Interprofessional collaboration in healthcare is a complex process influenced by various internal and external factors, leading to varying degrees of effectiveness among multidisciplinary teams [23,24]. Despite its importance, collaboration between general physicians and emergency department teams remains understudied [23]. Existing research often focuses narrowly on communication, overlooking the broader aspects of interprofessional collaboration. As a result, the gap between primary and secondary care remains largely unaddressed, particularly in healthcare systems with limited integration strategies to promote collaboration [23].

Systems and Structural Barriers

Workflow, Referral Issues and Time Pressure: A study by Janssens et al. highlights the challenges associated with permeable teams, particularly the issue of workflow interruptions [25]. Workflow interruptions are defined as unexpected suspensions of task performance or attentional focus, often resulting from rapid advancement of technology and fluid team structures [25-27]. These interruptions can significantly impact team functioning, including team meetings, and can arise from various sources such as external phone calls, technological malfunctions, and physical disruptions [25,27]. Understanding the effects of interruptions on team communication is crucial, as it can hinder a team’s ability to diagnose problems and develop interventions [25]. Effective team communication is essential for team performance, and interruptions can harm team functioning, leading to miscommunication, dissatisfaction, stress, delays, and distractions [27]. Furthermore, interruptions can affect not only the interrupted team member but also other members of the team, highlighting the need for further research on managing interruptions in team settings [25,28]. The way health care is organised can affect relationships between physicians. Mutual understanding, shared goals, and balanced power are core components of workflow in systemic collaboration between emergency physicians and family medicine doctors. Structural barriers arise in the absence of clear roles, strong leadership, supportive work environments, and accessible out-of-hours services [23]. The structure of after-hours care services can also impact emergency room utilisation [23,29]. Fragmented systems hinder team coordination. Competition between private GPs and emergency doctors can disrupt cooperation. Ambiguity in responsibilities leads to inefficiencies in collaborations. High emergency rooms use strain care coordination [23]. Also, a structure where patients can access emergency room services directly without a referral from a general physician could further disrupt the collaboration between emergency and family physicians [23]. Better teamwork between general and emergency practitioners, such as integration strategies to align both roles for seamless care delivery, is essential to improve care and control costs [30,31].

Institutional Culture, Documentation, and Follow-up Mechanisms: The relationship between general physicians and emergency department teams is heavily influenced by the way healthcare is organised and structured. External factors, such as policies and regulations, impact collaboration; lack of effective information exchange and active patient participation constitute a barrier between emergency medicine and family physicians [23]. Emergency physicians (EPs) and primary care physicians (PCPs) face significant challenges in communicating effectively. A fragmented and frustrating process with multiple pages and lengthy waits for callbacks hinders timely communication. Calls frequently interrupt patient care, heightening the risk of medical errors and unsettling patients [19]. Smooth transitions from emergency departments (ED) to primary care (PC) providers are crucial for ensuring high-quality, safe, and cost-effective patient care [19,21,32]. 

However, follow-up rates vary significantly due to several factors. Barriers to follow-up include financial constraints, in which patients may struggle with costs associated with follow-up care. With reference to patient perceptions, patients may not see the need for follow-up care or may not prioritise it [33,34]. With regards to socioeconomic factors, minority groups, low-income patients, and those without regular primary care providers may face challenges in accessing follow-up care. On the other hand, enablers of follow-up, which include health insurance, can facilitate access to follow-up care. Also, patients with a regular primary care provider are more likely to follow up due to established relationships. Clear communication between ED and PC providers can improve follow-up rates, classed as effective communication [19,34].

Emotional and Relational Dimensions

Stories of Success or Failure in Collaboration: A case in a study by Chipidza et al. highlights the benefits of collaborative care in managing patients with complex substance use disorders [35]. The case of a patient with intravenous drug abuse illustrates effective doctor-patient interaction. Despite multiple hospitalizations for complications, the multidisciplinary approach involving medical and addiction teams helped to address the substance use disorder. This collaborative approach enabled the team to recognize and address the patient’s desire to change his behaviour, facilitated through motivational interviewing [35]. By involving the substance abuse team, the medical team demonstrated a willingness to utilize available resources to overcome the challenges of treating a complex condition. This approach fostered a sense of trust and loyalty between the patient and his healthcare providers, ultimately contributing to more effective care [35].

A successful collaboration in patient care involves several key elements. Firstly, a comprehensive assessment is crucial to recognise the patient’s underlying condition, allowing healthcare providers to understand the complexity of their needs. Specialised expertise is then utilised through consultations with relevant teams, such as substance abuse specialists, to ensure that all aspects of the patient’s care are addressed. Patient-centered care is also vital, and techniques like motivational interviewing help empower patients to take an active role in their treatment. Ultimately, building a therapeutic relationship founded on trust and empathy is essential for fostering a supportive and non-judgmental environment, leading to more effective care and better patient outcomes [35,36]. This case study underscores the importance of interdisciplinary collaboration in addressing the complex needs of patients with substance use disorders. 

Another interesting case in the same article by Chipidza et al. highlights challenges in collaborative care, particularly in doctor-patient communication. The case of a 75-year-old woman diagnosed with gastric carcinoma highlights the significance of effective communication in healthcare. The stark contrast between the oncologist’s empathetic approach and the surgeon’s blunt delivery underscores the need for a patient-centered approach. While the oncologist’s clear explanation helped establish trust, the surgeon’s failure to consider the patient’s emotional state led to distress and a breakdown in their relationship. By prioritizing empathy, clarity, and patient involvement, healthcare providers can build trust and foster more positive outcomes, ultimately creating more supportive and collaborative relationships with their patients [35].

The Emotional Impact on Patients and Providers: Collaborative care is a multifaceted intervention that integrates frontline social work, nursing, and physician providers to address the complex physical, emotional, and social needs of patients [37]. Given its alignment with principles of trauma-informed care, including patient-provider collaboration, prevention of repeat trauma, and comprehensive mental and physical healthcare. They hypothesized that collaborative care may be particularly beneficial for patients with a history of violent victimization [37,38]. To investigate this hypothesis, they conducted an exploratory secondary data analysis of collaborative care randomized clinical trials involving patients who presented with traumatic physical injury at a Level 1 trauma center in Washington state between 2006 and 2009 [37]. The analysis utilised random-effect linear regression models to estimate the moderating effects of multiple violent trauma histories on the collaborative care intervention’s impact on short form-36 mental component summary (MCS) and physical component summary (PCS) T-scores over time [37]. The results of the analysis revealed that collaborative care significantly improved follow-up MCS scores among patients who experienced three to four types of violent victimization in their lifetime. Notably, the intervention effects on MCS scores at both three and six-month follow-up were clinically stronger for patients with a history of multiple victimization experiences. 

In contrast to findings for MCS scores, analysis did not reveal any significant differences in intervention effects on PCS scores between patients with and without a history of violent victimization. This suggests that collaborative care may have a more pronounced impact on mental health outcomes for patients with a history of multiple types of violent victimization [37]. Overall, findings suggest that collaborative care may be a promising approach to delivering trauma-informed mental healthcare to patients with histories of multiple types of violent victimization. By integrating patient-provider collaboration, prevention of repeat trauma, and collaborative mental and physical healthcare, collaborative care may offer a valuable framework for addressing the complex needs of this patient population [37].

Patient Outcomes and Perceptions

How Collaborative Practices Shape Patients' Perceptions of Care Quality: A study conducted by Morgan et al. (2020) found that patients reported a significantly enhanced quality of care when receiving interprofessional care [39]. This care model not only fostered improved relationships with healthcare professionals but also promoted patient-centered qualities, including more positive attitudes, increased attention, and greater availability among healthcare providers [39]. Notably, patients felt more integrated into the care team, suggesting a shift towards a more collaborative approach [39]. Furthermore, patients attributed their experiences with interprofessional collaboration in primary care to increased participation, improved self-management of their conditions, enhanced self-efficacy, and greater engagement in their care. These interconnected concepts are linked to a spectrum of improved health behaviours and outcomes, underscoring the potential benefits of interprofessional care in primary settings [39,40].

Shaw SN, in her study, elucidated the concept of “good care” in the context of interprofessional collaboration [41]. According to the findings, patients perceived high-quality care as being rooted in a strong patient-professional relationship that enables seamless access to and communication among healthcare professionals [41]. Notably, patients endorsed the “three A’s”, availability, affability, and ability, as the most valued characteristics of a healthcare team, a sentiment often echoed by family physicians [41]. While the care delivery model exhibited several patient-centered dimensions, the study also revealed that healthcare professionals occasionally struggled to find common ground with patients. In response to these challenges, interprofessional care was initiated as a strategy to navigate these conflicts and improve patient care [41,42]. Some duties showed that patients with chronic conditions such as diabetes valued the collaborative relationships they developed with their healthcare providers [43]. By working closely with physicians and diabetes educators in familiar primary care settings, participants felt included in their care and were able to acquire knowledge and set goals in a comfortable and convenient environment [43-45]. Many patients also preferred one-on-one sessions, which allowed for personalised attention. The study highlighted the importance of the patient-provider relationship, characterised by respect, supportive interactions, and facilitation of patient engagement [43]. Notably, the supportive environments created by diabetes educators fostered trusting relationships, which in turn enhanced patients’ motivation to improve their self-care practices [43,46].

Interprofessional collaboration (IPC) has been posited to positively influence patient outcomes by enhancing communication, defining shared goals, and leveraging the expertise of multiple professionals [47]. This, in turn, is expected to improve care coordination and continuity. However, implementing IPC is complex and requires healthcare professionals to adopt novel approaches that optimize resource utilization while cost-effectively delivering comprehensive primary care, while some studies have demonstrated the benefits of IPC across various healthcare settings, others have reported limited or inconclusive evidence, highlighting the need for further investigation [47]. As interest in IPC in primary care continues to grow, a deeper understanding of its effectiveness, implementation processes, and underlying mechanisms is essential to inform best practices and optimize patient care [47,48].

Similarly, a study by Martin-Rodriguez et al. (2008) examined the outcomes of interprofessional collaboration for hospitalised cancer patients. The results indicate that higher intensity of interprofessional collaboration is associated with improved patient satisfaction, reduced uncertainty, and enhanced pain management [49]. However, the study also found that the degree of collaboration did not have a significant impact on the length of hospital stay, suggesting that while interprofessional collaboration can improve certain aspects of patient care, its effects may be nuanced and variable across different outcomes [49].

Discussion

Research suggests that collaborative care has made notable strides, as exemplified in Belgium. A study by Karam et al. highlights significant developments in the country’s healthcare landscape since 2003 [23]. Publicly funded organized duty centers, including out-of-hours centers and walk-in clinics, have emerged through initiatives led by general physician circles. These centers have improved working conditions for physicians by streamlining on-call duties and reducing home visits. Strategically positioning some centres near hospital emergency services has yielded benefits, including reduced overcrowding [23]. By providing primary care to patients without access to a general physician or those whose physician is unavailable, these centres decrease unnecessary emergency department visits. Furthermore, they enable emergency departments to refer patients with primary care needs, optimizing resource allocation and care delivery [23,50]. The Belgian model showcases the potential for collaborative care innovations to enhance healthcare efficiency and effectiveness. Research has consistently demonstrated that a sense of control is significantly correlated with various positive health outcomes, including enhanced pain tolerance, expedited recovery from illness, reduced tumor growth, and improved daily functioning [51-53]. Furthermore, studies have reported that individuals with a greater sense of control exhibit better psychological adjustments and mental health [51,54]. This, in turn, can lead to reduced hospital stays, lower medical costs, and fewer referrals. Patient-centered encounters have been shown to yield better outcomes for both patients and doctors, with increased satisfaction reported by both parties. Notably, patient-doctor agreement on treatment plans and follow-up care is strongly linked to improved recovery rates [51]. Effective communication between patients and doctors is also crucial, as patients who report good communication are more likely to be satisfied with their care, share relevant information for accurate diagnosis, adhere to treatment plans, and follow advice [51]. Moreover, highly engaged patients tend to exhibit better health outcomes, including controlled high-density lipoprotein cholesterol and triglyceride levels, reduced smoking and obesity rates, and increased adherence to preventive care measures such as pap smears and mammograms [55]. Engaged patients also tend to have better-managed depression, fewer emergency visits, and reduced hospitalization rates [55]. These findings underscore the importance of patient-centered care and effective patient-doctor communication in achieving optimal health outcomes. When considering the limitations and future directions of research on the doctor-patient relationship, it is notable that a significant portion of the existing literature relies heavily on patient satisfaction and adherence as primary metrics for evaluating efficacy [51]. However, the generalizability of these findings is often constrained by factors such as sample size and the representativeness of the study population [51]. To better understand the causal relationships between various factors and doctor-patient communication, future research should prioritize investigations that focus on tightly defined and homogenous case mixes, allowing for more nuanced explorations of satisfaction and its determinants [51].

Effective care planning that aligns with patients' values, priorities, and preferences can significantly reduce the burden of self-management and minimize the risk of unwanted treatments [56-58]. This alignment is particularly crucial for older adults with multiple chronic conditions, who often face unique challenges such as cognitive decline, mental health issues, social isolation, complex medication regimens, and limited health literacy [56,59]. Research has consistently shown that patient-centered care can be difficult to deliver, with discrepancies often arising between patients' and physicians' priorities. Patients tend to focus on symptom management and immediate needs, while physicians often prioritize long-term risks and disease management [56,60]. To bridge this gap, physicians frequently engage in compromise with their patients, underscoring the importance of dedicated time and effort in clinical encounters to tailor care plans that meet the complex needs of these patients [56]. By prioritizing patient-centered care, healthcare providers can better support older adults with multimorbidity in achieving their health goals and improving their overall well-being. 

According to Montori et al. 2023, patient stories are often underrepresented in collaborative care. To address this gap, the authors propose several strategies to enhance patient-clinician collaboration and shared decision-making (SDM). One approach involves utilizing home visits, photographs, and narrative accounts of daily living to develop a deeper understanding of the social and economic challenges patients face. The "My Healthcare, My Life" conversation tool is also suggested as a means to foster a joint understanding of patients' lives and circumstances [5,61]. Moreover, incorporating patient stories into care planning can be particularly valuable in critical care situations, enabling patients, families, and clinicians to make informed decisions about life-support interventions. The authors also emphasize the importance of using SDM tools that facilitate effective communication of evidence and numerical risk information. These tools should be user-friendly, non-intrusive, and supportive of the patient-clinician conversation [5]. Some SDM tools are effective in randomized trials and are available for free use. Additionally, the "teach-back" method can be employed to verify that patients and clinicians have a mutual understanding of the information shared during consultations [5].

Research directions

Further research should prioritize mixed-methods studies that capture both quantitative and qualitative aspects of collaborative care. This approach will provide a more comprehensive understanding of its effectiveness and identify areas for improvement. Longitudinal designs can also help examine the long-term impact of collaborative care on patient outcomes, healthcare utilisation, and cost-effectiveness. Comparative effectiveness research is needed to determine the most effective collaborative care models and strategies for different patient populations and settings. Moreover, research should focus on patient-centered outcomes, such as patient satisfaction, quality of life, and functional status, to ensure that care is tailored to meet the needs and priorities of patients.

Practice implications

In terms of practice, healthcare providers should receive training and education on collaborative care principles, communication skills, and teamwork. Co-designed care pathways, developed in collaboration with patients, families, and healthcare providers, can help ensure that care plans are patient-centered and effective. Effective communication tools, such as shared decision-making frameworks and patient portals, can facilitate collaboration and patient engagement. Team-based care models, which bring together healthcare providers from different disciplines, can provide comprehensive and patient-centered care. Continuous quality improvement initiatives should be implemented to monitor and improve the effectiveness of collaborative care models.

Implementation considerations

To successfully implement collaborative care models, stakeholder engagement is crucial. This involves engaging patients, families, and healthcare providers in the design and implementation of care models. Change management strategies can support the adoption of new care models and practices. Technology, such as electronic health records and telehealth platforms, can be leveraged to support collaborative care and improve communication among healthcare providers and patients. Performance metrics should be developed and tracked to evaluate the effectiveness of collaborative care models and identify areas for improvement. Finally, sustainability planning is essential to ensure the long-term viability of collaborative care models and practices. By prioritising these research and practice recommendations, healthcare systems can promote more effective and patient-centered collaborative care.

## Conclusions

The collaboration between emergency medicine and family medicine physicians is essential for holistic, patient-centered care, as it bridges the gap between acute crisis management and ongoing health management. By integrating their unique expertise, these teams can improve continuity of care, address complex patient needs more comprehensively, and enhance overall health outcomes-ensuring patients receive seamless, coordinated treatment across acute and long-term settings. Such collaboration fosters shared understanding of patient histories, reduces fragmented care, and supports tailored interventions, ultimately leading to more effective, personalized healthcare delivery and better patient experiences.
